# Finding small molecules for the ‘next Ebola’

**DOI:** 10.12688/f1000research.6181.2

**Published:** 2015-07-07

**Authors:** Sean Ekins, Christopher Southan, Megan Coffee

**Affiliations:** 1 Collaborations in Chemistry, 5616 Hilltop Needmore Road, Fuquay-Varina, NC, 27526, USA; 2 Collaborative Drug Discovery, 1633 Bayshore Highway, Suite 342, Burlingame, CA, 94010, USA; 3 IUPHAR/BPS Guide to PHARMACOLOGY, Centre for Integrative Physiology, University of Edinburgh, Hugh Robson Building, Edinburgh, EH8 9XD, UK; 4 Center for Infectious Diseases and Emergency Readiness, University of California at Berkeley, 1918 University Ave, Berkeley, CA, 94704, USA

**Keywords:** ebola, text mining, open science, outbreak, patents, databases, box model

## Abstract

The current Ebola virus epidemic may provide some suggestions of how we can better prepare for the next pathogen outbreak. We propose several cost effective steps that could be taken that would impact the discovery and use of small molecule therapeutics including: 1. text mine the literature, 2. patent assignees and/or inventors should openly declare their relevant filings, 3. reagents and assays could be commoditized, 4. using manual curation to enhance database links, 5. engage database and curation teams, 6. consider open science approaches, 7. adapt the “box” model for shareable reference compounds, and 8. involve the physician’s perspective.

## Introduction

The current Ebola virus (EBOV) epidemic points to opportunities for preparing for the next pathogen outbreak or newly identified infectious disease. While control measures and therapeutic strategies have certainly been learned from past outbreaks ^1^, they may be insufficient to control a new one. Given the rapid evolution of viruses and the inexorable global increase in human mobility, this quote from a recent Nature editorial seems prescient “ *because one thing is clear: whether it is Ebola virus, another filovirus or something completely different, there will be a next time*” ^2^. For comparison we can also look at infectious diseases we do have treatments for but still need to improve and/or circumvent drug resistance. For example in our experience in tuberculosis and malaria the patchiness of explicit chemistry connectivity between papers, patents and database entries impedes progress. In the case of EBOV with less than 1500 papers in PubMed and only 25 crystal structures in the Protein Data Bank (at the time of writing), mechanistic aspects that could open the way for therapeutic developments are still not elucidated. Like others ^3– 5^ our focus is on small molecule interventions ^6– 8^ and we have therefore considered various steps that might help prepare for future pathogens as follows:

## Text mine the literature

The highest quality and density of information about pathogens resides in peer reviewed publications, patents and databases. In recent years, text mining in general and natural language processing in particular, has become the method of choice for the extraction and collation of facts from document corpora ^9^. This could thus have a rapid payoff in mining for the similarities and differences between emergent versus known pathogens. In the case of EBOV we have immediately found antiviral medicinal chemistry basic recall searches (i.e. not authentic text mining) had specificity challenges. This was also observed with synonyms for EBOV, related isolates and phylogenetic neighbors (e.g. Marburg virus) ^10^. A corollary of this is that full text of at least EBOV papers could be released for text mining outside pay-walls, by agreement with publishers. Several publishers have agreed to make publications freely accessible ( http://www.oxfordjournals.org/en/our-journals/medicine-and-health/ebola.html; http://www.springer.com/biomed/virology/spotlight+on+ebola?SGWID=0-1771314-0-0-0) so this would not be too difficult.

## Patent assignees and/or inventors should openly declare their relevant filings

Patents contain more published medicinal chemistry data than papers ^11^. For example nearly 200 WO patents for HIV protease inhibitors can be retrieved by a simple word search. This information source also presents a paradox in being, on the one hand, difficult to extract structured data from because of varying degrees of obfuscation, but on the other, full-text is easier to access than papers. In addition, not only does PubChem contain over 18 million structures from patents but also SureChEMBL now automatically extracts the chemistry from newly published filings within days. Preliminary queries indicate the patent corpus covering direct EBOV entry or replication inhibitor chemistry (or for host processing proteases as targets) is small but would nonetheless be very important to access. The only way to make retrieval rapid and complete is for assignees to openly declare their relevant published patent titles and numbers that are inevitably missed by keyword searching. In addition extraction would be much more effective if they re-surfaced the data to make it more accessible. This could be as simple as just uploading an Excel sheet to Figshare (or other open repository) with a few hundred rows of structures (linked to PubChem CIDs where these are already out there), activity values and short assay descriptions, rather than leaving the community to grapple with a hundred page PDF. We realize this is unprecedented but as a type of emergency response has to be considered. Assignee organizations should also encourage their inventors to do exactly this. Logically, another ‘precedent breaker’ can be considered, namely that applicants publish or surface their anti-pathogen patent results effectively the day after filing. This may sound scary to some, but IP rights are conserved while, in community terms, 18 months are cut off the “information shadow” phase. Another move in the right direction has been shown by the World Intellectual Property Organization Re:Search Consortium. Their initiative to open up patents for neglected tropical disease research could also be extended to cover filoviruses and other viruses ( http://www.wipo.int/research/en/about/index.html).

## Reagents and assays could be commoditized

Research can be accelerated by the collaborative exchange of assay reagents and protocols between teams and this has other positive consequences beyond just speed. Crucially, it contributes to inter-lab reproducibility if assays are made robust enough to be transferred. In addition, structure activity relationship (SAR) results will have reduced variance and will thus be more comparable between laboratories. This reciprocity becomes particularly valuable if a pharmaceutical company or other organization (Molecular Libraries Screening Centre, or Euro Screen for example) engages to run a high throughput screen. Consequently, multiple collaborators can pick up the baton of analog expansion of confirmed hits via the same standardized assay. A good example in the EBOV case is a recent publication of PDB structures for small molecules that bind the filovirus VP35 protein and inhibit its polymerase cofactor activity ^12^. Supplies of assay-ready VP35 (even from a reagent vendor) would thus be valuable to expand take-up by more screening centers.

## Using manual curation to enhance database links

Once a search of various publications and databases is complete one should be able to navigate reciprocally from a molecule identifier to a structure or from a target to modulating chemistry. This is not always the case, for example where pharmaceutical company lead structures are obfuscated ^13^. A relevant example of useful linkages is exemplified by “ *Pyridinyl imidazole inhibitors of p38 MAP kinase impair viral entry and reduce cytokine induction by Zaire ebolavirus in human dendritic cells*” ^14^. It certainly helped that SB202190 is PubChem positive as CID 5353940 (NJNKPVPFGLGHPA-UHFFFAOYSA-N). This is well-linked as a kinase inhibitor but not directly to these recent EBOV results. The essential role of facilitating such bioactive chemistry linkage is taken by curated databases ^15– 17^. These not only curate literature-extracted activity results into structured records but also merge this connectivity into PubChem. The overall data findability/linkability could be further enhanced if the MeSH system could fast-track new EBOV-specific indexing and shorten the lag time.

## Engage database and curation teams

Data mining is expedited by activity results and associated chemical structures being captured in databases. But the time to publish a paper and index the contents into structured records may take years. However, major chemistry resources such as PubChem ^18^, ChEMBL ^19^ and ChemSpider ^20^ are all willing (by prior arrangement) to take direct submissions (e.g. from EBOV screening teams) possibly even pulled directly from electronic lab notebooks (ELNs) along with the crucial metadata. The same approach of data generators actively engaging with speeding up and improving the transfer of their own results into databases applies equally to the sequence side of things. For EBOV the bioinformatics and genomics communities appear demonstrably ahead of the game compared to the medicinal chemistry community. For example the ViralZone in Europe and the US Virus Pathogen Resource were quickly established as integrated knowledge portals ^21^ ( http://www.viprbrc.org/brc/home.spg?decorator=vipr). Significantly, the related problems of terminology mapping for curators and retrieval specificity for users is already being addressed ^22^. What might be less well known is that authors submitting sequences can apply this rule by engaging directly with database staff (e.g. including feedback for MeSH indexing) to ensure the rapid and precise annotation of new virus entries.

## Consider Open Science approaches

If Open Science approaches are considered, the advantages of this to pathogen drug discovery (including the abrogation of intellectual property generation) primary data sharing can then become instantaneous and global ^23^. The consequent shortening of drug research stages can be dramatic. For example an InChIkey surfaced from an ELN (or other open instantiations such as Wikis or Figshare ( http://figshare.com/)) means that chemistry becomes findable within hours of Google indexing ^24^. It also frees teams from the ‘tyranny of novelty’ where new leads can be rationally optimized from pre-existing ones. In contrast the conventional IP-centric research model not only includes the years of delay to prepare a paper (i.e. 18 months after a patent application) but, even then, not all relevant antiviral chemistry flows from papers into public databases. This can also expedite the free exchange of reagents and protocols. The Open Science model can also leverage the “wisdom of the crowd” such that a global volunteer cadre of experienced chemists (industry and academic) can immediately participate both in SAR interpretation and in the design cycle. We would also suggest open sharing not only of small molecules or data sets from relevant assays, but also of a range of predictive (and sharable) models or hypotheses that can be used for virtual screening. Any SAR data from the literature can be mined to understand physicochemical properties or molecular features important for antiviral activity. Such ligand-based computational models in turn could then be used for searching additional libraries of compounds (e.g. pharma companies might even implement this on their complete proprietary screening collections and share the results). Curated data sets could be used to construct “whole cell” virus specific machine learning models, similar to those for *Mycobacterium tuberculosis* ^25^. Computational algorithms like Connectivity Map ^26^, SEA ^27^ and others could be implemented to enable fast querying of the data so that the most similar virus to an unknown could be found, and from there the most active compounds selected. Software like Euretos BRAIN, could be used to mine relationships between different biological terms and molecules that can then be used for target inference ^28^.

## Adapt the “box” model for shareable reference compounds

The idea here is to create an openly available diverse set of compounds that are not likely to yield false positives, aggregators or other undesirable structural types commonly termed PAINS ^29^. These “box” compounds would possess known antiviral and anti-pathogen activity plated out for wide availability. The notable precedents here are the MMV Malaria Box ( http://www.mmv.org/malariabox), the forthcoming Pathogen Box ( http://pathogenbox.org/) and the NIH Clinical Collection ( http://www.nihclinicalcollection.com/). These could be hosted by a third party on behalf of NIAID, CDC etc. This model is flexible in terms of multiple “boxes” being possible. For example sets of ~1400 screening-ready FDA approved drugs (that physicians would have ready access to), would be the first logical pass for repurposing investigations. The next “box” could include the ~8000 structures in PubChem that include an International Nonproprietary Name (INN) designation and therefore in most cases have clinical testing. The advantages of what we can call ‘virtuous circularity’ of connectivity, apply exactly in this case. Specifically the major chemical databases can ensure a) they tag availability (i.e. a retrievable flag “this compound is in free box Y”, b) the publications, patents and historical assay results are linked to the same entries and crucially c) new results (with appropriate provenance) from users of box compounds are promptly added back into the database records. By logical extension the computational modelling efforts can then loop through more rounds of improvement, new testing, leading to better hits that are put back into the ‘box’.

## Involve the physician’s perspective

As we are seeing with EBOV, clinicians and healthcare providers are the first line of defense for the rest of the world. They are also at the greatest risk from the pathogen themselves. They are also clearly in the best position to decide how to treat their patients; the steps above should result in treatments that can actually be obtained ^8^ and tolerated by the patient. Physicians with experience of treating infectious diseases could be engaged to group treatments as 1. drugs which they would use in patients who are very ill; 2. those drugs which would be of concern as they may do more harm than good and 3. those drugs which might be used regardless (to explore if effective). In the case of a virulent pathogen with only palliative treatment options, from a physician’s perspective, anything that reduces mortality (even slightly) is crucial or which may even have other clinical endpoints (such as reduced hospitalization or reduced symptoms) even if mortality isn’t affected. Another way to think of this is to increase the number of patients that can be treated.

In conclusion, what we are seeing now has a precedent in other viruses we were not “expecting” (e.g. HIV). Even decades on we have combination therapies to control the disease but no cure or vaccine. For EBOV we have had nearly 40 years to prepare. The cost effective suggestions above could be implemented to prepare for when the next new pathogen arrives, otherwise we will be in the same situation again. We propose that as new pathogens are identified we should be able to rapidly identify new antiviral drugs as well as establish where approved drugs that physicians have experience with, can be effective. This approach could be applicable to other infectious diseases beyond those which we currently know.
