# Supplementary material for: Finding small molecules for the ‘next Ebola’
Source: F1000Res. 2015 Jul 7;4:58. Originally published 2015 Feb 27. [Version 2] doi: 10.12688/f1000research.6181.2 (PMC4406187; doi:10.12688/f1000research.6181.2)
Supplement: Peer review history

# Peer review: Referee response for version 2

**DOI:** 10.5256/f1000research.7247.r9539

**Published:** 2015-07-17

**Authors:** Qiaoying Zeng

**Affiliations:** 1 College of Veterinary Medicine, Gansu Agricultural University, Lanzhou, China

The authors have addressed my concerns, and I am happy to confirm my ‘Approved’ status.

I have read this submission. I believe that I have an appropriate level of expertise to confirm that it is of an acceptable scientific standard.

# Peer review: Referee response for version 1

**DOI:** 10.5256/f1000research.6626.r8318

**Published:** 2015-04-14

**Authors:** Martin Zacharias

**Affiliations:** 1 Physics Department, Technical University Munich, Garching bei München, Germany

The recent Ebola virus outbreak came as a surprise and the authors suggest possible steps to improve the situation in case of another Ebola epidemic outbreak. The main focus is on cost effective easy implementable steps that could be taken to accelerate drug development or other therapeutic approaches. I think the suggested strategies and cost effective approaches are relevant not only in case of Ebola but could also be useful in case of other infectious diseases. The paper is well and clearly written and of interest for a broad readership in the area of pharmaceutical research, medicine and other biomedical disciplines.

I have read this submission. I believe that I have an appropriate level of expertise to confirm that it is of an acceptable scientific standard.

# Response

**Published:** 2015-07-03

**Authors:** Sean Ekins

**Affiliations:** Collaborations in Chemistry, USA

Thank you for this review.

# Peer review: Referee response for version 1

**DOI:** 10.5256/f1000research.6626.r8258

**Published:** 2015-04-13

**Authors:** Qiaoying Zeng

**Affiliations:** 1 College of Veterinary Medicine, Gansu Agricultural University, Lanzhou, China

The article proposed an integrative strategy in anticipation of the next outbreak of an emerging or reemerging infectious disease like Ebola. The authors suggested seven cost effective steps that help to establish a synergistic mechanism for a fast discovery of small molecule therapeutics.  Under this mechanism, the information and data from publications, patents, and database, even some reagents and assays in different labs, could be shared instantaneously and efficiently among scientific and clinical communities around the globe. The direct experience of physicians is also a great plus. These cost effective approaches could be implemented to prepare in advance for the next pathogen outbreak or newly identified infectious disease that is definite no matter when or where it starts. The article is interesting and will be beneficial for an efficient battle against the future new outbreaks of infectious diseases.

Minors:  Some sentences are confused and needed to be more concise or clarified:

1. In the case of EBOV we immediately found antiviral medicinal chemistry basic recall searches (i.e. not authentic text mining) had specificity challenges, even just associated with synonyms for EBOV, related isolates and phylogenetic neighbors (e.g. Marburg virus).
2. The advantages of this to pathogen drug discovery (including the abrogation of intellectual property generation) primary data sharing can then become instantaneous and global.

I have read this submission. I believe that I have an appropriate level of expertise to confirm that it is of an acceptable scientific standard.

# Response

**Published:** 2015-07-03

**Authors:** Sean Ekins

**Affiliations:** Collaborations in Chemistry, USA

Thank you for these suggestions which have now been addressed in the latest version.
